# Factors associated with physical, psychological and social frailty among community-dwelling older persons in Europe: a cross-sectional study of Urban Health Centres Europe (UHCE)

**DOI:** 10.1186/s12877-021-02364-x

**Published:** 2021-07-12

**Authors:** Lizhen Ye, Liset E. M. Elstgeest, Xuxi Zhang, Tamara Alhambra-Borrás, Siok Swan Tan, Hein Raat

**Affiliations:** 1 Department of Public Health, Erasmus MC - University Medical Center Rotterdam, P.O. Box 2040, 3000 Rotterdam, CA The Netherlands; 2 Center for Healthy Aging and Development Studies, National School of Development, Peking University, Beijing, 100871 China; 3 Polibienestar Research Institute - Universitat de València ES, 29 46022 Valencia, Spain

**Keywords:** Frailty, Physical frailty, Psychological frailty, Social frailty, Urban health Centres Europe (UHCE) study, Sex, Household composition, Alcohol use, Smoking, Medication use

## Abstract

**Background:**

Frailty is an age-related condition resulting in a state of increased vulnerability regarding functioning across multiple systems. It is a multidimensional concept referring to physical, psychological and social domains. The purpose of this study is to identify factors (demographic characteristics, lifestyle factors and health indicators) associated with overall frailty and physical, psychological and social frailty in community-dwelling older people from five European countries.

**Methods:**

This cross-sectional study used baseline data from 2289 participants of the Urban Health Center European project in five European countries. Multivariable logistic regression models were used to assess associations of the factors with overall frailty and the three frailty domains.

**Results:**

The mean age was 79.7 (SD = 5.7). Participants who were older, were female, had secondary or equivalent education, lived alone, not at risk of alcohol use, were less physically active, had multi-morbidity, were malnourished or with a higher level of medication risk, had higher odds of overall frailty (all *P* < 0.05). Age was not associated with psychological and social frailty; sex was not associated with social frailty; smoking and migration background was not associated with overall frailty or any of its domains. There existed an interaction effect between sex and household composition regarding social frailty (*P* < 0.0003).

**Conclusions:**

The present study contributed new insights into the risk factors for frailty and its three domains (physical, psychological and social frailty). Nurses, physicians, public health professionals and policymakers should be aware of the risk factors of each type of frailty. Furthermore, examine these risk factors more comprehensively and consider overall frailty as well as its three domains in order to further contribute to decision-making more precisely on the prevention and management of frailty.

**Trial registration:**

The intervention of the UHCE project was registered in the ISRCTN registry as ISRCTN52788952. The date of registration is 13/03/2017.

**Supplementary Information:**

The online version contains supplementary material available at 10.1186/s12877-021-02364-x.

## Background

With the rapid expansion of the ageing population [1], the number of older adults with frailty is also increasing. Frail persons need extra medical attention and are high users of community resources, hospitalization and nursing homes [2], thereby placing further pressure on health care pressure and increasing the financial burden on the health system [3].

Frailty is defined as an age-related condition characterized by an increased state of vulnerability in functioning across multiple physiological systems [4]. Frail individuals live with an increased risk of adverse health outcomes, including falls [5], fractures [6], disability [4] and morbidity when exposed to a stressor. According to a broader definition, frailty is a multidimensional concept referring to not only physical but also psychological and social domains [7–9]. Fried et al. developed the concept of frailty from a physical aspect, which has been widely used worldwide [4]. The Frailty Index is extracted from the psychological aspect and consists of a count of impairments in various areas, such as mood, cognition and incontinence [10]. Social frailty is defined as a state of being at risk of losing (or having already lost) resources that are essential for meeting one or more basic social demands [11].

As suggested by Cook et al. (2017), due to the multidimensional nature of frailty, the combination of physical, psychological and social frailty is more likely to contribute to disability and mortality than physical, psychological or social frailty alone [6]. In contrast to this multidimensional approach, some of previous studies only focused on one of the domains of frailty: physically [12], psychologically [13] or socially [11]. However, uncovering the potential pathways of frailty in combination with its three domains is essential and could increase our understanding of frailty from a more comprehensive perspective. It is critical to develop effective prevention strategies for frailty to reduce its impact at the level of both the individual and the health system, and, consequently, to build an age-friendly world.

An initial step to develop prevention strategies for frailty is to explore factors associated with frailty, which include the identification of groups at risk of becoming frail [13]. Some studies have only focused on the concept of frailty but have not explored the associated factors [4, 14, 15]. Various other studies have focused on analyzing factors associated with frailty. However, their findings have been inconsistent and sometimes even contradictory. For instance, age, sex, education, smoking and alcohol intake were reported to be associated with frailty [1, 16]. Yet Buttery et al. (2015) found no significant association between sex and frailty [17]. Ye, B. et al. (2018) found that smoking was not associated with frailty among adults aged over 60 in Shanghai, China [18]. Furthermore, a 2-year follow-up study among European community-dwelling persons over 55 years found that greater alcohol consumption was actually associated with a lower risk of developing frailty [19]. These inconsistencies show that more research is needed on these factors and on other factors that might be related to frailty, such as physical activity [20] household composition [21], multi-morbidity [22].

The objective of the current study is to identify the factors associated with overall frailty, as well as with physical, psychological and social frailty, among community-dwelling older people from five European countries. The factors included in the study are demographic characteristics, lifestyle factors and health indicators.

## Methods

### Study design and setting

This cross-sectional study used the baseline data of the Urban Health Centres Europe (UHCE) project, which aimed to promote healthy aging of older persons. The project was conducted in five European cities (Greater Manchester, the United Kingdom; Pallini, Greece; Rijeka, Croatia; Rotterdam, The Netherlands; and Valencia, Spain) [23].

A preventive, multidimensional assessment was performed to assess frailty, healthy lifestyle, appropriate medication use, level of independence, fall risk, loneliness level, health-related quality of life and care use. There were 6472 older people aged 70 years and older who lived independently were invited, and a total of 2325 participants aged 70 years and older who lived independently were enrolled in 2015 [23]. Data were collected using a self-report questionnaire that included the UHCE assessment (described above), outcome and other measures. All the baseline data were collected in May 2015. More details on the study design have been described in detail elsewhere [23–25].

Participants with missing data on age and sex (*n* = 6) and on overall frailty or on the three domains of frailty (physical, psychological and social domain; *n* = 30) were excluded, resulting in a sample of analysis of 2289 participants.

### Frailty

Frailty was assessed by the Tilburg Frailty Indicator (TFI), a validated questionnaire to identify frailty among the older population in primary care [26]. The TFI assesses frailty from a bio-psycho-social structure [27]. A previous study has confirmed [28] that the TFI is a reliable and valid instrument to measure frailty in community-dwelling older citizens in five European countries: Spain, Greece, Croatia, the United Kingdom and the Netherlands. It contains 15 self-reported questions that focus on three domains: physical frailty (8 items, score range 0–8), psychological frailty (4 items, score range 0–4) and social frailty (3 items, score range 0–3) [27, 29]. The overall frailty score equals the sum of the three domain scores (score range 0–15) [27]. Participants with a total score of at least 5 on overall frailty were categorized as being frail. The cut-off points for physical, psychological and social frailty were 3, 2, and 2, respectively [30]. People can be frail on one or more domains simultaneously, while overall non-frail people can be frail with regard to one of the separate domains.

### Potential factors associated with frailty

All factors were measured by a self-report questionnaire [23]. Demographic characteristics included age (in years), sex (male/female), country of residence (United Kingdom, Greece, Croatia, The Netherlands and Spain), migration background (yes/no), household composition (dichotomized as living with others/living alone), and education level. A participant was classified as having a migration background when his/her country of residence differed from his/her country of birth. Education level had three categories according to the International Standard Classification of Education (ISCED): primary or less (ISCED 0–1), secondary or equivalent (ISCED 2–5), and tertiary or higher (ISCED 6–8) [31].

Lifestyle factors included alcohol risk, physical activity and smoking. Alcohol risk was assessed by three items of the Alcohol Use Disorders Identification Test (AUDIT-C) [24], resulting in a score ranging from 0 (lowest risk) to 12 (highest risk). The variable was dichotomized (≥4 in males and ≥ 3 in females) to indicate presence/absence of alcohol risk (yes/no), i.e. a risk that drinking is affecting the participant’s health and safety [32]. Smoking was dichotomized as being a current smoker (yes/no) [23, 33, 34]. The frequency of physical activity was measure by a question from the Frailty Instrument of the Survey of Health, Ageing and Retirement in Europe (SHARE-FI) [35]. Participants were dichotomized into being engaged in physical activity that requires low or moderate energy either once a week or less, or more than once a week [33].

Health indicators included the presence of multi-morbidity (yes/no), medication risk and malnutrition (yes/no). Multi-morbidity was measured as having had experienced or currently having at least 2 of 14 common chronic conditions [36], including heart attack, high blood pressure or hypertension, high blood cholesterol, stroke or cerebral vascular disease, diabetes or high blood sugar, chronic lung disease, asthma, arthritis, osteoporosis, cancer or malignant tumor, stomach or duodenal ulcer or peptic ulcer, Parkinson’s disease, cataract, and hip fracture or femoral fracture. Medication risk was measured with 10 items of the Medication Risk Questionnaire (MRQ-10), resulting in a score ranging from 0 to 10 (higher scores refer to lower levels of appropriate medication use) [37]. Malnutrition was assessed with the Short Nutrition Assessment Questionnaire 65+ (SNAQ-65+) [38], which is a screening tool for determining undernutrition among community-dwelling persons aged 65 and over [39]. SNAQ-65+ consists of a question on unintentional weight loss in the past 6 months, mid-upper arm circumference (MUAC) and questions on appetite and functional status. Malnutrition was defined if weight loss happened (person lost 6 kg or 13lbs or more during the last 6 months, or 3 kg or 6½ lbs. or more during the last month) or if a MUAC was < 25 cm.

### Statistical analyses

Descriptive statistics were used to describe the characteristics of the participants. Continuous variables were summarized as means and standard deviation (SD), and categorical variables were displayed as frequencies and percentages. Characteristics of participants were compared by T-test for continuous variables and by means of chi-square tests for categorical variables for frail and non-frail groups. Multivariable logistic regression models were used to assess associations of the factors with overall, physical, psychological and social frailty. Odds ratios and 95% confidence intervals (95% CI) were calculated for each factor. *P*-values of 0.05 or lower were considered to be statistically significant. Finally, in order to assess effect-modification by age, sex, country and education level, we assessed interactions between these four variables and the factors in the associations of the studied factors with frailty and the three domains of frailty. According to the guidelines of Knol and VanderWeele [40], Bonferroni correction for multivariable logistic regression was applied for analysis of the interaction items (*P* = 0.05/152 = 0.0003). All analyses were conducted in the Statistical Package for Social Sciences (SPSS), version 25 for Windows (IBM SPSS Statistics for Windows, IBM Corp).

## Results

### Characteristics of the participants

Table 1 presents the general characteristics of the participants (*n* = 2289). The mean age was 79.7 (SD 5.7) years, and 60.2% were women. A total of 1267 (55.4%) participants were frail. Compared with non-frail participants, frail participants were older and more often female, were more often from Greece and Croatia, more often had a lower educational level, lived less often with others, were less often at risk for alcohol use, engaged less often in physical activity, more often had multi-morbidity, had lower levels of appropriate medication use, and were more often malnourished (all *P* < 0.05).

**Table 1:** Baseline characteristics of community-dwelling older persons of the Urban Health Centres Europe for total study sample (*n* = 2289) and according to overall frailty

	Total (***n*** = 2289)	Frailty		
		No (***n*** = 1022, 44.6%)	Yes (***n*** = 1267, 55.4%)	***P***-value
**Age (years)**	79.7 ± 5.7	78.8 ± 5.4	80.5 ± 5.7	< 0.001^a^
**Sex, female**	1379(60.4%)	503(49.3%)	876 (69.4%)	< 0.001^b^
**Country**				
Spain	500 (21.8%)	252 (24.7%)	248 (19.6%)	< 0.001^b^
Greece	363 (15.9%)	133 (13.0%)	230 (18.2%)	
Croatia	490 (21.4%)	126 (12.3%)	364 (28.7%)	
The Netherlands	373 (16.3%)	213 (20.8%)	160 (12.6%)	
United Kingdom	563 (24.6%)	298 (29.2%)	265 (20.9%)	
**Migration background, yes**	194 (8.50%)	81 (7.90%)	113 (8.90%)	0.396^b^
**Education level**				
Primary or less	621 (27.5%)	245 (24.3%)	376 (30.0%)	< 0.001^b^
Secondary or equivalent	1430 (63.2%)	646 (64.0%)	784 (62.6%)	
Tertiary or higher	211 (9.30%)	119 (11.8%)	92 (7.30%)	
**Household composition, living alone**	876 (38.4%)	288 (28.3%)	588 (46.6%)	< 0.001^b^
**Alcohol risk, yes**	582 (26.8%)	340 (34.6%)	242 (20.3%)	< 0.001^b^
**Physical activity**				
More than once a week	1628 (71.8%)	883 (87.2%)	745 (59.4%)	< 0.001^b^
Once a week or less	640 (28.2%)	130(12.8%)	510(40.6%)	
**Smoking, yes**	175 (7.70%)	74 (7.30%)	101 (8.00%)	0.528^b^
**Multi-morbidity, yes**	2083(91.1%)	868 (85.1%)	1215 (95.9)	< 0.001^b^
**Medication risk (MRQ-10; score)**	4.40 ± 1.64	4.06 ± 1.51	4.67 ± 1.68	< 0.001^b^
**Malnutrition (SNAQ-65+), yes**	356 (15.6)	76 (7.5%)	280 (22.3%)	< 0.001 ^b^

Presented as mean ± SD or N (%); Significant *P*-values (< 0.05) in bold

Missing items: Age = 2; Sex =5; Education level = 27; Household composition =7; Alcohol risk =116; Physical activity =21; Smoking =5; Multi-morbidity =2; Medication risk = 26; Malnutrition = 12

*Abbreviations*: *SD* standard deviation, *MRQ-10* 10 items of the Medication risk questionnaire, *SNAQ-65+*, Short Nutritional Assessment Questionnaire 65 +

^a^ *P*-values based on independent T test

^b^ *P*-values based on chi-square test

Supplementary Table S1 shows the general characteristics for each of the three domains of frailty. Among the 2289 participants, 1243 (54.3%) were physically frail, 896 (39.1%) were psychologically frail, and 673 (29.4%) were socially frail. A total of 674 (29.4%) participants were not frail on any of the three domains, 703 (30.7%) participants were frail on one domain, 627 (27.4%) on two domains, and 285 (12.5%) on three domains.

#### Multivariable associations of potential factors with overall, physical, psychological and social frailty

Table 2 presents the multivariable logistic regression model on associations between the potential factors and overall frailty. Participants who were older, were women, lived alone, engaged in physical activities once a week or less, had with multi-morbidity, had a higher level of medication risk (i.e. lower levels of appropriate medication use), survived with malnourished had higher odds of being frail than those who were not (*P* < 0.001). Participants from Spain, Greece, Croatia, the UK had a higher odds of being frail than participants from the Netherlands (*P* < 0.001). Participants who completed a secondary or equivalent educational level (*P* < 0.05), but not tertiary level, had higher odds of being frail than those with a lower educational level. Participants at risk of alcohol use had lower odds of being frail than those not at risk (*P* < 0.05). Two factors, migration background and smoking, were not significantly associated with overall frailty.

**Table 2:** Multivariable associations between potential associated factors and overall frailty (*n* = 2289)

	Overall frailty	
	OR (95%CI)	***P***-value
**Age (years)**	**1.06 (1.04–1.08**)	**< 0.001**
**Sex (female vs. male)**	**2.20 (1.75–2.76)**	**< 0.001**
**Country**		**< 0.001**
Spain vs. the Netherlands	**1.93 (1.34–2.78)**	**< 0.001**
Greece vs. the Netherlands	**4.71 (3.11–7.13)**	**< 0.001**
Croatia vs. the Netherlands	**4.24 (2.94–6.12)**	**< 0.001**
United Kingdom vs. the Netherlands	1.19 (0.85–1.63)	0.323
**Migration background (yes vs. no)**	0.90 (0.61–1.32)	0.588
**Education level**		**0.027**
Secondary or equivalent vs. primary or less	**1.58 (1.05–2.37)**	**0.029**
Tertiary or higher vs. primary or less	1.10 (0.76–1.59)	0.631
**Household composition (living alone vs. living with others)**	**2.11 (1.68–2.66)**	**< 0.001**
**Alcohol risk (yes vs. no)**	**0.76 (0.61–0.96)**	**0.023**
**Physical activity (once a week or less vs. more than once a week)**	**3.71 (2.88–4.77)**	**< 0.001**
**Smoking (yes vs. no)**	1.37(0.93–2.01)	0.113
**Multi-morbidity (yes vs. no)**	**2.54 (1.69–3.81)**	**< 0.001**
**Medication risk (MRQ-10; score)**	**1.33 (1.24–1.42)**	**< 0.001**
**Malnutrition (SNAQ-65+; yes vs. no)**	**3.06 (2.22–4.22)**	**< 0.001**

*Abbreviations*: *OR* odds ratio, *CI* confidence interval, *MRQ-10* 10 items of the Medication risk questionnaire, *SNAQ-65+* Short Nutritional Assessment Questionnaire 65 +

Significant ORs and *P*-values (< 0.05) in bold

Multivariable model were used to analysis the associations between potential associated factors and overall frailty. All factors (e.g. demographic characteristics, lifestyle factors and health indicators) were included in one model. Nagelkerke R_1_^2^ = 0.37

Table 3 presents the multivariable logistic regression models on associations between the potential factors and the three domains of frailty. A higher age was associated with higher odds of being physically frail (*P* < 0.001) but not with being psychologically or socially frail. Compared with participants from the Netherlands, people from Greece and Croatia had a higher odds of being physical frail (*P* < 0.001), people from Spain, Greece and Croatia had a higher odds of being psychological frail (*P* < 0.001), and people from Spain and Greece had a higher odds of being social frail (*P* < 0.05). Compared with those with a lower educational level, participants who completed a secondary or equivalent educational level had higher odds of being physically (*P* < 0.001) and psychologically frail (*P* < 0.01), but not being with socially frail. Participants who lived alone had lower odds of being physical frail (*P* < 0.05) but higher odds of being social frail (*P* < 0.001) than participants who lived with others. Participants who were at risk of alcohol use were less likely to be physically frail (*P* < 0.01). Participants who engaged in physical activities once a week or less had higher odds of being physically (*P* < 0.001), psychologically (*P* < 0.001) and socially (*P* < 0.01) frail compare to more physically active participants. People with multi-morbidity had higher odds of being physically (*P* < 0.001) and socially (*P* < 0.05) frail than those without. Higher levels of medication risk (i.e. lower levels of appropriate medication use) were associated with higher odds of being physically (*P* < 0.001), psychologically (*P* < 0.001), and socially frail (*P* < 0.05). Participants who were malnutrition had higher odds of being physically (*P* < 0.001), psychologically (*P* < 0.001) and socially frail (*P* < 0.05) than those were not.

**Table 3:** Multivariable associations of potential associated factors with physical, psychological and social frailty (*n* = 2289)

	Physical frailty (***n*** = 1243 yes)		Psychological frailty (***n*** = 896 yes)		Social frailty (3 items, cutoff = 2) (***n*** = 673 yes)	
	OR (95%CI)	***P***-value	OR (95%CI)	***P***-value	OR (95%CI)	***P***-value
**Age (years)**	**1.06 (1.04–1.08)**	**< 0.001**	1.01 (0.99–1.03)	0.408	1.02 (0.10–1.04)	0.143
**Sex (female vs. male)**	**2.17 (1.73–2.72)**	**< 0.001**	**1.83 (1.47–2.29)**	**< 0.001**	0.91 (0.70–1.20)	0.507
**Country**		**< 0.001**		**< 0.001**		**< 0.001**
Spain vs. the Netherlands	1.40 (0.98–1.99)	0.067	**1.93 (1.34–2.78)**	**< 0.001**	**0.53 (0.35–0.81)**	**0.004**
Greece vs. the Netherlands	**2.20 (1.48–3.28)**	**< 0.001**	**5.35 (3.59–7.95)**	**< 0.001**	**1.84 (1.17–2.90)**	**0.009**
Croatia vs. the Netherlands	**3.19 (2.24–4.54)**	**< 0.001**	**3.92 (2.79–5.51)**	**< 0.001**	1.33 (0.91–1.94)	0.148
United Kingdom vs. the Netherlands	1.10 (0.80–1.51)	0.558	1.22 (0.87–1.71)	0.244	**0.52 (0.37–0.75)**	**< 0.001**
**Migration background (yes vs. no)**	1.05 (0.72–1.54)	0.792	0.98 (0.69–1.41)	0.922	0.88 (0.57–1.34)	0.550
**Education level**		**< 0.001**		**0.010**		**0.037**
Secondary or equivalent vs. primary or less	**2.12 (1.42–3.17)**	**< 0.001**	**1.85 (1.24–2.78)**	**0.003**	1.09 (0.68–1.75)	0.714
Tertiary or higher vs. primary or less	1.38 (0.96–1.99)	0.086	1.43 (0.98–2.07)	0.063	0.72 (0.47–1.11)	0.135
**Household composition (living alone vs. living with others)**	**0.80 (0.64–0.10)**	**0.048**	0.90 (0.72–1.12)	0.334	**15.54 (11.81–20.44)**	**< 0.001**
**Alcohol risk (yes vs. no)**	**0.67 (0.54–0.84)**	**0.001**	0.85 (0.67–1.07)	0.158	1.00 (0.76–1.31)	0.971
**Physical activity (once a week or less vs. more than once a week)**	**3.58 (2.81–4.57)**	**< 0.001**	**2.02 (1.63–2.50)**	**< 0.001**	**1.51 (1.17–2.00)**	**0.002**
**Smoking (yes vs. no)**	1.45 (0.99–2.11)	0.055	0.92 (0.63–1.34)	0.669	0.87 (0.57–1.35)	0.541
**Multi-morbidity (yes vs. no)**	**2.09 (1.41–3.08)**	**< 0.001**	1.32 (0.90–1.92)	0.155	**1.75 (1.06–2.88)**	**0.028**
**Medication risk (MRQ-10; scores)**	**1.35 (1.26–1.44)**	**< 0.001**	**1.15 (1.08–1.22)**	**< 0.001**	**1.1 (1.03–1.19)**	**0.005**
**Malnutrition (SNAQ-65+; yes vs. no)**	**2.53 (1.86–3.43)**	**< 0.001**	**1.76 (1.35–2.29)**	**< 0.001**	**1.21 (0.89–1.66)**	0.226

*Abbreviations*: *OR* odds ratio, *CI* confidence interval, *MRQ-10* 10 items of the Medication risk questionnaire, *SNAQ-65+* Short Nutritional Assessment Questionnaire 65 +

Significant ORs and *P*-values (< 0.05) in bold

Multivariable models were used to analysis the associations between potential associated factors with physical, psychological and social frailty

All factors (e.g. demographic characteristics, lifestyle factors and health indicators) were included in each model

Nagelkerke R_2_^2^ = 0.32 (physical frailty); Nagelkerke R_3_^2^ = 0.21 (psychological frailty), Nagelkerke R_4_^2^ = 0.39 (social frailty)

All *P*-values of the interaction analyses are presented in Supplementary Table S3. Notably, one statistically significant interaction was found: the interaction between sex and household composition regarding social frailty (*P* < 0.0003). Stratified analyses showed that the association between household composition (living alone) and social frailty was stronger among men than among women (Male: OR = 26.2, *P* < 0.05; Female: OR = 14. 9, *P* < 0.05).

## Discussion

We assessed demographic characteristics, lifestyle factors and health indicators that might associated with overall frailty as well as three domains of frailty within a diverse population group from five European countries. The present study confirms previous findings on association between factors (e.g. female sex, education level, country, physical activity, multi-morbidity, medication risk, and malnutrition) and frailty and its three domains. Remarkably, it shows that age was not associated with psychological and social frailty; sex was not associated with social frailty; people at risk of alcohol use had a lower risk of overall frailty and physical frailty; and smoking was not associated with frailty nor its three domains.

### Demographic characteristics

The present study confirms [41, 42] that overall frailty, and especially physical frailty, is highly associated with age. Remarkably, age was not associated with psychological or social frailty. Although age itself could be a risk factor for one’s physical condition due to human physiology, age may not necessarily be a specific risk factor for psychological and social frailty. For example, an older person might lose his or her spouse, then start to live alone and becomes isolated, which is an adverse life event that may negatively influence the psychological dimension of frailty. Moreover, if people cannot participate in social groups to the same extent as they had previously due to reasons independent of age, social resources that are essential for fulfilling their basic social needs may be lost. Consequently, this loss may lead to social frailty. These situations can happen at any stage of a person’s life, and are not by definition associated with older age. In this perspective, it is a specific adverse life event, rather than age, that may affect the psychological and social dimension of frailty. The age range of the participant is 70 to 102 y (the mean age is 79.7 ± 5.7), which is not a very diverse age population. A previous study illustrated that psychological frailty was affected by life events among community-dwelling persons aged 75 years and older [43]. People within this age range already experienced several life events. Their ability to cope with different situations, even the ability to recover from an adverse event, may be higher than in younger age. This may explain why age in itself was not to be a risk factor predictive of becoming psychologically or socially frail in our study.

Our results confirm previous findings [1, 44] that women, compared to men, have a relatively higher risk of having overall, physical and psychological frailty. Previous studies [41, 45] have suggested that older men have a greater likelihood of dying suddenly, while women more often show a steady decline, associated with an increase in co-morbidity and disability. Therefore, women might be frail more often, compared to men. Remarkably, in our study, sex was not associated with social frailty; this contradicts earlier findings [46, 47]. This non-consensus might be due to the different concepts of social frailty. In our study, three items were considered: living alone, missing having people around and receiving enough support from other people. However, social frailty is a relatively unexplored concept. To study the association between sex and social frailty, a more precise concept of social frailty and the developing pathways need to be explored in depth.

We found some differences regarding overall frailty and its three domains in the populations off Greece, Croatia and Spain, compared to the Netherlands. These differences could be explained by differences in socioeconomic, political and cultural backgrounds [48]. Advanced levels of democracy and egalitarian political traditions may contribute to the population health improvement of a country’s population and to a lower prevalence of frailty [49]. Further studies should be conducted to explore these differences between countries and to provide explanations for them.

In our study, migration background was not associated with overall frailty, nor with physical, psychological or social frailty. However, our study has a relatively low number of participants with a migrant background (*n* = 194), which might have reduced the power to detect such associations. To investigate the associations more comprehensively, we, therefore, recommend future studies with a larger number of participants from a migration background.

Our results show that people who completed secondary or an equivalent education have a relatively higher risk of overall, physical and psychological frailty. Education level was associated with frailty components, such as (instrumental) activities of daily living ((I)ADL), and self-rated health in several studies [48, 50]. Previous studies have concluded that people with a lower education level are, on average, frailer than people with a higher education level [51]. However, in our study, a tertiary or higher education level was not statistically significantly associated with overall frailty and its domains. It might be that the power of our study was too low to explore the association between educational level and frailty (211participants with tertiary or higher education).

We found that people who lived alone had a higher risk of overall frailty and social frailty, but a lower risk of physical frailty. People living alone had a lower risk of physical frailty might be because they were more likely to manage all the housework and other daily living tasks by themselves, thereby offering more opportunities to engage in physical activities. In line with this result, physical activities could contribute to reducing the risk among older people of being overall frailty as well as physical, psychological and social frailty. We found that the association between living alone and psychological frailty was not statistically significant. This finding can be explained by the fact that older people living alone may not be able to recognize mental health problems due to their social and financial vulnerability and the lack of proper formal/informal personal support. In light of this, it is possible that psychological frailty might also remain unrecognized. More studies are needed to clarify these findings.

With regard to social frailty, it should be noted that ‘living alone’ is one of the three items that defines social frailty in the Tilburg Frailty Indicator [26]. Because of this definition, the association between ‘household composition (i.e. living alone)’ and social frailty is artificially increased; therefore, we performed additional analyses with a definition of social frailty based on two items (excluding the item ‘living alone’). To define the dichotomous variable ‘social frailty-2 items’, we applied a cut-off score of 2 points as well as 1 point. With a cut-off of 2 points, Household composition-living alone was significantly associated with social frailty in the multivariable model (OR = 1.53, *P* < 0.01); with a cut-off of 1 point, this association was also significant (OR = 2.15, *P* < 0.001). So, ‘living alone’ is independently associated with social frailty. For example, after the loss of a partner and then living alone, the subsequent potential loss of social resources and activities may induce social frailty. Additionally, we made a multivariable model of the potential factors except ‘household composition-living alone’ and ‘social frailty-3 items’ (the original definition). This model showed that also age and sex were significantly associated with social frailty (*p* < 0.05). See Supplementary Table S2.

The impact of ‘living alone’ on social frailty might differ between women and men [11, 52] because of different ways of dealing with social situations. In both the original analyses and the additional analyses with a 2-item definition of social frailty, among men the association between living alone and social frailty was stronger than among women (see Supplementary Table S3). Further research is therefore needed to explore the differences between men and women regarding the impact of household composition on social frailty.

### Lifestyle factors

Remarkably, the results showed that people ‘at risk of alcohol use’ had a relatively lower risk of overall frailty and physical frailty; moreover, there was no association with psychological and social frailty. These findings was in contrast with a previous research [53]. An explanation for this might be that alcohol may often be consumed in a moderate and socially accepted way; accordingly, moderate consumption may facilitate social bonding [54]. It has been illustrated that increasing social contact and social support have an association with better health behavior [55, 56], which further results in better health outcomes: reduce the chance of being ill and positively influence the overall frailty and its three domains [50, 57, 58]. While this study did not study on the amount or frequency of alcohol intake, further studies should explore levels of alcohol intake in relation to frailty and its three domains.

We found that people who engaged in physical activities only once a week or less were more likely to be frail (both overall and its three domains). These results are in line with the results of a previous study [33]. Previous studies have concluded that physical activities could help older people realize that their bodies can still function well, increase connections with other people [59] and then decrease the occurrence of depression or depressive symptoms [60] and further improve their emotional well-being [61]. Under these mechanisms, physical activities could contribute to a lower risk of overall frailty, and physical, psychological and social frailty among older people.

Smoking was not significantly associated with frailty and its three domains in this study. However, as was stated in previous studies, smoking can damage a range of tissues and organs [62], and it is associated with diseases such as peripheral vascular disease [63], coronary heart disease [64], cancer [65], respiratory diseases [66], multiple sclerosis [67]. All these adverse effects and diseases can negatively influence the physical, psychological and social health of smokers and may lead to frailty [68, 69]. A potential reason for this might be that we dichotomized smoking into ‘current smoker’ and ‘not current smoking’. It did not consider the amount of smoking or former smoking. Further research is needed to investigate the association between smoking and frailty, including considering the amount of smoking and the smoking history.

### Health indicators

In line with previous studies, we found that people who have experienced or currently have at least 2 out of 14 common chronic conditions were associated with a higher risk of being overall frail, and physically and socially frail. Previous studies found that chronic diseases are considered to be major determinants of frailty [66]. A particular chronic disease could contribute to a specific component of frailty and initiate or worsen frailty [66]. For example, heart failure and other morbidities accelerate muscle loss, leading to sarcopenia [70], which further results in rapid functional decline. As has been established, functional decline is closely associated with frailty [4]. Higher levels of medication risk were associated with higher risks of being overall frail and physically, psychologically and socially frail, which has been confirmed by other studies [71, 72]. Ageing is associated with an increased prevalence of non-communicable diseases and an increased need for various medications. As a result, an increased risk of inappropriate medication use could occur. Participants who were reported to be malnourished were more likely to be overall frail, and physically, psychologically and socially frail, which is consistent with previous studies [73, 74]. Unintentional weight loss is one of the items defining frailty [75]. So, there exist overlap between frailty and sarcopenia [76]. Muscle mass is low in sarcopenia and poor nutrition may further accelerate loss of muscle mass. This may result in decreasing physical functioning, and further causing adverse outcomes such as falls, infections and pressure sores [74]. The accumulation of adverse health conditions can result in frailty [4, 5].

The presence of an interaction effect between sex and household composition on social frailty, indicates that the associations of all studied factors on social frailty varies between different sex as a function of household composition. As previous study have shown [11], after losing their partner, women more frequently living without a spouse than men. So women are more likely to be engaged in physical activity, but less likely be recognized from potential psychological risks. In addition, women have traditionally played the role of caregiver, may have better life skills, and may not seek care that may be helpful [77]. It could result in potential undiscovered health problems. However, all these findings could not fully explain the interaction effect of sex and household composition on social frailty. Further studies are needed to clarify this finding.

#### Limitations and strengths

Our results should be interpreted in the light of some limitations. First, due to the cross-sectional study design, we cannot infer causality. Second, persons were excluded if they lacked the basic knowledge of the local language or if they were not expected to be able to make an informed decision regarding participation in the project. Some of excluded persons might have had a migration background, some might not have been well educated, some might have had a severe health problem. Therefore, our findings may have under-estimated frailty at the population level. Third, we used dichotomous outcome measures of frailty, which may have resulted in loss of information. However, this increases the understanding for practice.

The present study has several notable strengths. First, it is among the few studies that has explored factors of frailty from a multidimensional perspective. We used a validated instrument to consider frailty broadly from the physical, psychological and social domains. Second, the target population is from five diverse European cities. This provides information on this study of a coordinated preventive care approach in various European settings.

## Conclusions

In conclusion, the present study contributed new insights into the risk factors for frailty and its three domains (physically, psychologically and socially). Age, sex, country, education level, household composition, alcohol risk, physical activity, multi-morbidity, medication risk, and malnutrition were associated with overall frailty and some of them with physical, psychological and/or social frailty. Smoking and migration background were not associated with overall frailty and its three domains. Nurses, physicians, public health professionals and policymakers should be aware of the risk factors of each type of frailty. Furthermore, examine these risk factors more comprehensively and consider overall frailty as well as its three domains in order to further contribute to decision-making more precisely on the prevention and management of frailty.

## Supplementary Information

**Additional file 1.**

## Data Availability

The datasets analysed during the current study are not publicly available due to privacy/ethical restrictions but are available from the corresponding author on reasonable request.

## Abbreviations

EU: European Union
UHCE: Urban Health Centres Europe
TFI: Tilburg Frailty Indicator
MRQ-10: 10 items of Medication Risk Questionnaire
SNAQ-65+: Short Nutrition Assessment Questionnaire 65+
MUAC: Mid-upper arm circumference
SD: Standard deviation
OR: Odds ratios
95% CI: 95% confidence intervals
IADL: Instrumental Activities of daily living
UK: United Kingdom
NL: Netherlands
