# Supplementary material for: Factors associated with physical, psychological and social frailty among community-dwelling older persons in Europe: a cross-sectional study of Urban Health Centres Europe (UHCE)
Source: BMC Geriatr. 2021 Jul 12;21:422. doi: 10.1186/s12877-021-02364-x (PMC8274028; doi:10.1186/s12877-021-02364-x)
Supplement: Supplementary file 1 — Additional file 1. [file 12877_2021_2364_MOESM1_ESM.docx]

**Supplementary Table S1 Participants characteristics according to three domains of frailty**

|  | **Physical frailty** | | | **Psychological frailty** | | | **Social frailty** | | |
| --- | --- | --- | --- | --- | --- | --- | --- | --- | --- |
|  | **No (1046, 45.7%)** | **Yes (1243, 54.3%)** | **P-value** | **No (1393, 60.9%)** | **Yes (896, 39.1%)** | **P-value** | **No (1616, 70.6%)** | **Yes (673, 29.4%)** | **P-value** |
| **Age (years)** | 78.7±5.5 | 80.5±5.6 | **<0.001^a^** | 79.8±5.6 | 79.6±5.8 | 0.530^a^ | 79.2±5.5 | 80.9±5.8 | **<0.001^a^** |
| **Sex (female)** | 541 (51.8%) | 838 (67.6%) | **<0.001^b^** | 764 (54.9%) | 615 (68.9%) | **<0.001^b^** | 893 (55.4%) | 486 (72.2%) | **<0.001^b^** |
| **Country** |  |  |  |  |  |  |  |  |  |
| Spain | 253 (24.2%) | 247 (19.9%) | **<0.001^b^** | 318 (22.8%) | 182 (20.3%) | **<0.001^b^** | 400 (24.8%) | 100 (14.9%) | **<0.001^b^** |
| Greece | 165 (15.8%) | 198 (15.9%) |  | 157 (11.3%) | 206 (23.0%) |  | 248 (15.3%) | 115 (17.1%) |  |
| Croatia | 124 (11.9%) | 366 (29.4%) |  | 217 (15.6%) | 273 (30.5%) |  | 316 (19.6%) | 174 (25.9%) |  |
| the Netherlands | 205 (19.6%) | 168 (13.5%) |  | 287 (20.6%) | 86 (9.60%) |  | 245 (15.2%) | 128 (19.0%) |  |
| United Kingdom | 299 (28.6%) | 264 (21.2%) |  | 414 (29.7%) | 149 (16.6%) |  | 407 (25.2%) | 156 (23.2%) |  |
| **Migration background (yes)** | 78 (7.50%) | 116 (9.30%) | 0.114^b^ | 118 (8.50%) | 76 (8.50%) | 1.000^b^ | 134 (8.30%) | 60 (8.90%) | 0.626^b^ |
| **Education level** |  |  |  |  |  |  |  |  |  |
| primary or less | 256 (24.8%) | 365 (29.7%) | **<0.001^b^** | 339 (24.6%) | 282 (31.9%) | **<0.001^b^** | 435 (27.2%) | 186 (28.1%) | 0.543^b^ |
| secondary or equivalent | 645 (62.6%) | 785 (63.8%) |  | 885 (64.2%) | 545 (61.7%) |  | 1008 (63.0%) | 422 (63.7%) |  |
| tertiary or higher | 130 (12.6%) | 81 (6.60%) |  | 154 (11.2%) | 57 (6.40%) |  | 156 (9.80%) | 55 (8.30%) |  |
| **Household composition (living alone)** | 376 (36.0%) | 500 (40.4%) | **0.035^b^** | 540 (38.9%) | 336 (37.6%) | 0.526^b^ | 360 (22.3%) | 516 (77.1%) | **<0.001^b^** |
| **Alcohol risk (yes)** | 352 (35.2%) | 230 (19.6%) | **<0.001^b^** | 404 (30.4%) | 178 (21.1%) | **<0.001^b^** | 433 (28.2%) | 149 (23.4%) | **0.020^b^** |
| **Physical activity** |  |  |  |  |  |  |  |  |  |
| more than once a week | 905 (87.3%) | 723 (58.7%) | **<0.001^b^** | 1091 (78.8%) | 537 (60.7%) | **<0.001^b^** | 1199 (74.9%) | 429 (64.2%) | **<0.001^b^** |
| once a week or less | 132 (12.7%) | 508 (41.3%) |  | 293 (21.2%) | 347 (39.3%) |  | 401 (25.1%) | 239 (35.8%) |  |
| **Smoking (yes)** | 77 (7.40%) | 98 (7.90%) | 0.645^b^ | 111 (8.00%) | 64 (7.20%) | 0.468^b^ | 124 (7.70%) | 51 (7.60%) | 0.943^b^ |
| **Multi-morbidity (yes)** | 895 (85.7%) | 1188 (95.6%) | **<0.001^b^** | 1244 (89.4%) | 839 (93.7%) | **<0.001^b^** | 1447 (89.6%) | 636 (94.6%) | **<0.001^b^** |
| **Medication risk (MRQ-10; scores)** | 4.03 ± 1.53 | 4.71 ± 1.67 | **<0.001^a^** | 4.29 ± 1.58 | 4.58 ±1.72 | **<0.001^a^** | 4.33 ± 1.59 | 4.56± 1.75 | **0.004^a^** |
| **Malnutrition (SNAQ-65+) (yes)** | 82 (7.90%) | 274(22.2%) | **<0.001^b^** | 166(12.0%) | 190(21.4%) | **<0.001^b^** | 232(14.4%) | 124(18.5%) | **0.014^b^** |

Note: Presented as mean ± SD or N(%); Significant P-values (<0.05) in bold.

Missing items: Sex =5; Education level =27; Household composition =7; Alcohol risk =116; Physical activity =21; Smoking =5; Multi-morbidity =2.

Abbreviations: SD, standard deviation; MRQ-10, 10 items of the Medication risk questionnaire; SNAQ-65+, Short Nutritional Assessment Questionnaire 65+

a P-values based on independent t test

b P-values based on chi-square test

**Supplementary Table S2 Additional analysis on associations of potential associated factors with social frailty (n=2289).**

|  | **Social frailty**  **(2 items, cutoff=2)**  **(n=265yes)** | | **Social frailty**  **(2 items, cutoff=1)**  **(n=1179yes)** | | **Social frailty**  **(without Household composition,**  **3 items, cutoff=2)**  **(n=673yes)** | |
| --- | --- | --- | --- | --- | --- | --- |
|  | **OR (95%CI)** | **P-value** | **OR (95%CI)** | **P-value** | **OR (95%CI)** | **P-value** |
| **Age (years)** | 0.99 (0.96-1.02) | 0.551 | 1.02 (1.00-1.04) | 0.057 | **1.06 (1.04-1.08)** | **<0.001** |
| **Sex (female vs. male)** | 1.09 (0.78-1.52) | 0.609 | 0.99 (0.80-1.21) | 0.884 | **2.21 (1.77-2.76)** | **<0.001** |
| **Country** |  | **<0.001** |  | **<0.001** |  | **<0.001** |
| Spain vs. the Netherlands | 0.56 (0.33-0.96) | 0.036 | 0.93 (0.67-1.30) | 0.683 | **0.46 (0.32-0.67)** | **<0.001** |
| Greece vs. the Netherlands | 1.60 0.95-2.69 | 0.079 | **2.97 (2.05-4.31)** | **<0.001** | **1.09 (0.74-1.60)** | 0.664 |
| Croatia vs. the Netherlands | **1.73 1.11-2.72** | **0.016** | **1.86 (1.36-2.54)** | **<0.001** | 0.97 (0.70-1.34) | 0.856 |
| United Kingdom vs. the Netherlands | **0.44 0.26-0.75** | **0.002** | **0.72 (0.54-0.96)** | **0.024** | **0.69 (0.51-0.95)** | **0.021** |
| **Migration background (yes vs. no)** | 0.93 0.55-1.57 | 0.788 | 0.92 0.65-1.30 | 0.623 | 0.95 (0.66-1.36) | 0.760 |
| **Education level** |  | **0.041** |  | 0.058 |  | **0.049** |
| secondary or equivalent vs. primary or less | 1.58 0.87-2.85 | 0.131 | **1.48 (1.02-2.14)** | **0.039** | 1.00 (0.67-1.50) | 0.999 |
| tertiary or higher vs. primary or less | 0.95 0.55-1.66 | 0.865 | 1.20 (0.80-1.56) | 0.524 | 0.73 (0.50-1.05) | 0.086 |
| **Household composition (living alone vs. living with others)** | **1.53 1.12-2.10** | **0.007** | **2.15 (1.75-2.64)** | **<0.001** | / | / |
| **Alcohol risk (yes vs. no)** | 0.95 0.67-1.35 | 0.776 | 0.95 (0.77-1.18) | 0.639 | 0.97 (0.76-1.22) | 0.779 |
| **Physical activity (once a week or less vs. more than once a week)** | **1.51 1.11-2.04** | **0.008** | **1.65 (1.34-2.04)** | **<0.001** | **1.25 (1.004-1.56)** | **0.046** |
| **Smoking (yes vs. no)** | 0.91 0.53-1.56 | 0.738 | 1.01 (0.71-1.43) | 0.973 | 1.18(0.81-1.71) | 0.398 |
| **Multi-morbidity (yes vs. no)** | 1.35 0.73-2.49 | 0.337 | 1.15 (0.82-1.61) | 0.423 | **1.70 (1.10-2.63)** | **0.018** |
| **Medication risk (MRQ-10; scores)** | **1.71 1.21-2.40** | **0.020** | **1.08 (1.02-1.14)** | **0.012** | **1.12 (1.05-1.19)** | **<0.001** |
| **Malnutrition (SNAQ-65+; yes vs. no)** | **1.11 1.02-1.21** | **0.002** | **1.36 (1.05-1.76)** | **0.020** | 1.02 (0.79-1.34) | 0.861 |

Abbreviations: OR=odds ratio; CI=confidence interval;

MRQ-10, 10 items of the Medication risk questionnaire; SNAQ-65+, Short Nutritional Assessment Questionnaire 65+.

Significant ORs and P-values (<0.05) in bold.

Multivariable models were used to analysis the associations between potential associated factors with physical, psychological and social frailty.

All factors (e.g. demographic characteristics, lifestyle factors and health indicators) were included in each model.

**Supplementary Table S3 P-values of the interaction terms added to the multivariable logistic regression models on associations of factors with overall frailty and three domains of frailty among 2289 participants of the UHCE study.**

| **NO.** | **Interaction Items** | **Frailty** | **Physical frailty** | **Psychological frailty** | **Social frailty**  **(3 item, cutoff=2)** | Social frailty  (2 item, cutoff=1) | Social frailty  (without Household composition, 3 item, cutoff = 2) |
| --- | --- | --- | --- | --- | --- | --- | --- |
|  |  | **P-value** | **P-value** | **P-value** | **P-value** | P-value | P-value |
| 1 | Age*Sex | 0.175 | 0.516 | 0.051 | 0.313 | 0.262 | 0.371 |
| 2 | Age*Country | 0.625 | 0.713 | 0.345 | 0.395 | 0.958 | 0.356 |
| 3 | Age*Education level | 0.574 | 0.286 | 0.853 | 0.063 | 0.215 | 0.249 |
| 4 | Age*Migration background | 0.424 | 0.114 | 0.616 | 0.989 | 0.914 | 0.843 |
| 5 | Age*Household composition | 0.250 | 0.764 | 0.281 | 0.787 | 0.928 | / |
| 6 | Age*Alcohol risk | 0.142 | 0.374 | 0.919 | 0.077 | 0.053 | 0.039 |
| 7 | Age*Physical activity | 0.029 | 0.182 | 0.736 | 0.344 | 0.665 | 0.784 |
| 8 | Age*Smoking | 0.254 | 0.921 | 0.424 | 0.177 | 0.394 | 0.270 |
| 9 | Age*Multi-morbidity | 0.913 | 0.315 | 0.680 | 0.151 | 0.813 | 0.140 |
| 10 | Age*Medication risk | 0.993 | 0.810 | 0.204 | 0.700 | 0.832 | 0.323 |
| 11 | Age*Malnutrition | 0.190 | 0.840 | 0.379 | 0.081 | 0.044 | 0.066 |
| 12 | Sex*Country | 0.201 | 0.820 | 0.173 | 0.022 | 0.025 | 0.016 |
| 13 | Sex*Education level | 0.039 | 0.396 | 0.007 | 0.240 | 0.724 | 0.531 |
| 14 | Sex*Migration background | 0.655 | 0.850 | 0.663 | 0.270 | 0.325 | 0.203 |
| 15 | **Sex*Household composition** | 0.693 | 0.111 | 0.846 | **< 0.0003** | **< 0.0003** | / |
| 16 | Sex*Alcohol | 0.713 | 0.840 | 0.011 | 0.539 | 0.666 | 0.321 |
| 17 | Sex*Physical activity | 0.686 | 0.652 | 0.451 | 0.345 | 0.996 | 0.524 |
| 18 | Sex*Smoking | 0.188 | 0.174 | 0.075 | 0.445 | 0.181 | 0.117 |
| 19 | Sex*Multi-morbidity | 0.970 | 0.362 | 0.301 | 0.013 | 0.162 | 0.031 |
| 20 | Sex*Medication risk | 0.993 | 0.693 | 0.440 | 0.048 | 0.037 | 0.080 |
| 21 | Sex*Malnutrition | 0.121 | 0.137 | 0.005 | 0.576 | 0.761 | 0.475 |
| 22 | Country*Education level | 0.072 | 0.608 | 0.572 | 0.159 | 0.081 | 0.106 |
| 23 | Country*Migration background | 0.435 | 0.834 | 0.750 | 0.501 | 0.939 | 0.237 |
| 24 | Country*Household composition | 0.003 | 0.447 | 0.070 | 0.013 | 0.003 | / |
| 25 | Country*Life style-alcohol | 0.309 | 0.294 | 0.795 | 0.573 | 0.228 | 0.369 |
| 26 | Country*Physical activity | 0.153 | 0.772 | 0.077 | 0.812 | 0.385 | 0.911 |
| 27 | Country*Life style-smoking | 0.731 | 0.212 | 0.351 | 0.156 | 0.235 | 0.175 |
| 28 | Country*Multi-morbidity | 0.823 | 0.579 | 0.527 | 0.688 | 0.134 | 0.861 |
| 29 | Country*Medication risk | 0.612 | 0.630 | 0.309 | 0.760 | 0.512 | 0.014 |
| 30 | Country*Malnutrition | 0.892 | 0.856 | 0.402 | 0.065 | 0.051 | 0.058 |
| 31 | Education level*Migration background | 0.860 | 0.520 | 0.320 | 0.584 | 0.138 | 0.244 |
| 32 | Education level*Household composition | 0.076 | 0.564 | 0.049 | 0.056 | 0.005 | / |
| 33 | Education level*Alcohol risk | 0.098 | 0.057 | 0.820 | 0.205 | 0.293 | 0.135 |
| 34 | Education level*Physical activity | 0.172 | 0.182 | 0.345 | 0.706 | 0.601 | 0.543 |
| 35 | Education level*Smoking | 0.400 | 0.770 | 0.528 | 0.110 | 0.915 | 0.369 |
| 36 | Education level*Multi-morbidity | 0.292 | 0.121 | 0.462 | 0.507 | 0.511 | 0.404 |
| 37 | Education level*Medication risk | 0.783 | 0.434 | 0.685 | 0.500 | 0.894 | 0.327 |
| 38 | Education level*Malnutrition | 0.600 | 0.137 | 0.045 | 0.715 | 0.783 | 0.746 |

P-values were derived by separately adding the interaction terms to the multivariable logistic regression models for socio-demographic characteristics and overall frailty and three domains among 2289 participants of the UHCE study. Bonferroni correction for multivariable logistic regression was applied (P=0.05/38*4 = 0.0003)
